# 24-Hour Movement Behaviour and Health Awareness as Possible Predictors of Infertility-Related Quality of Life

**DOI:** 10.3390/jcm14186552

**Published:** 2025-09-17

**Authors:** Viktória Prémusz, Réka Kovács, Eszter Skriba, Gábor Szmatona, Zoltán Tándor, Alexandra Makai, Pongrác Ács, Kálmán Kovács, Ákos Várnagy, Ilona Veres-Balajti

**Affiliations:** 1 Institute of Physiotherapy and Sport Sciences, Faculty of Health Sciences, University of Pécs, H-7621 Pécs, Hungary; kovreka07@gmail.com (R.K.); alexandra.makai@etk.pte.hu (A.M.); pongrac.acs@etk.pte.hu (P.Á.); 2 Physical Activity Research Group, János Szentágothai Research Centre, University of Pécs, H-7624 Pécs, Hungary; 3 National Laboratory on Human Reproduction, University of Pécs, H-7622 Pécs, Hungary; kovacs.kalman@pte.hu (K.K.); varnagy.akos@pte.hu (Á.V.); 4 Doctoral School of Health Science, Faculty of Health Science, University of Pécs, H-7621 Pécs, Hungary; skriba.eszter@edu.pte.hu (E.S.); szmatona.gabor@edu.pte.hu (G.S.); tz@med.unideb.hu (Z.T.); 5 Dunamenti REK Reproduction Centre, H-1035 Budapest, Hungary; 6 Assisted Reproduction Centre, Clinical Centre, University of Debrecen, H-4032 Debrecen, Hungary; 7 Department of Obstetrics and Gynaecology, Medical School, University of Pécs, H-7624 Pécs, Hungary; 8 Directorate for Human Reproduction, National Directorate General for Hospitals, H-1125 Budapest, Hungary; 9 Department of Physiotherapy, Institute of Health Sciences, Faculty of Health Sciences, University of Debrecen, H-4028 Debrecen, Hungary; balajti.ilona@etk.unideb.hu

**Keywords:** infertility, quality of life, health literacy, fertility awareness, physical activity, 24-h movement behaviour

## Abstract

**Background/Objectives:** Infertility imposes substantial psychosocial burdens on affected individuals, often resulting in a decline in quality of life comparable to that experienced in chronic diseases. Exploring lifestyle and health awareness-related factors is essential to develop complex, multidisciplinary approaches. This study investigated the associations between the components of 24-h movement behaviour (physical activity, sedentary lifestyle, sleep), health literacy, fertility awareness, and general and infertility-specific quality of life. Additionally, the study assessed whether these factors could predict quality of life outcomes in women living with infertility. **Methods:** A cross-sectional study was conducted using questionnaire-based data collection in four fertility centres in Hungary. The convenience sample included 361 women aged 18–45 years with a documented infertility diagnosis. Validated questionnaires were used to assess health literacy (BRIEF), fertility awareness (FAS), physical activity (GPAQ-H), sleep quality (AIS), and quality of life (WHOQOL-BREF and FertiQoL). Data analysis included Kolmogorov–Smirnov tests, Spearman correlations, and generalised linear modelling (GLM), with statistical significance set at *p* < 0.05. **Results:** Based on the FAS, 77.8% of participants (*n* = 274) self-reported being adequately informed; however, objective knowledge scores accounted for only 48.5% of the possible total, indicating limited knowledge. Fertility awareness positively correlated with recreational physical activity (ρ = 0.156; *p* = 0.003). Recreational physical activity showed low but significant positive associations with all quality-of-life dimensions (e.g., psychological well-being: r = 0.177; *p* ≤ 0.002), whereas sedentary time was negatively associated with psychological well-being (r = −0.109) and social relationships (r = −0.118). Sleep duration correlated positively while sleep quality problems correlated negatively with FertiQoL scores (r = −0.339; *p* ≤ 0.001). Better sleep quality, lower sedentary time, and higher health literacy were positive predictors of infertility-specific quality of life, whereas higher fertility awareness showed a paradoxical adverse effect. **Conclusions:** These findings highlight the role of 24-h movement behaviour and health awareness in improving quality of life among women with infertility. The study supports the need for tailored, multi-component lifestyle interventions to promote physical, mental, and psycho-social well-being.

## 1. Introduction

One in six people globally—approximately 17.5% of the population—experience infertility at some point in their lives, according to the World Health Organization’s data [1,2]. This high prevalence highlights infertility as a significant global health challenge, affecting individuals across all income levels and regions. Beyond its medical implications, infertility imposes substantial psychosocial burdens, including elevated stress, social stigma, and negative impacts on mental health [3]. In some regions, infertility ranks as the third most distressing health condition among the affected age group, following cancer and cardiovascular diseases [2].

Multiple factors influence the outcomes of assisted reproductive technologies (ART), including age, sperm quality, fertilisation rate, embryo quality, the number of embryos transferred, and endometrial thickness [4]. Recent studies highlight the importance of biological aspects, such as environmental pollution, macro- and trace elements or early biomarkers of oxidative stress [5,6,7,8,9,10].

However, infertility management should not be limited to medical interventions alone, as improving the quality of life (QoL) of affected individuals requires medical and psychosocial support [11,12,13,14,15,16,17,18,19,20,21,22]. Given these complexities, it is essential to develop integrative treatment approaches beyond standard medical assessment. This includes the implementation of adjunctive lifestyle interventions, nutritional supplementation, and psychosocial support strategies. Such approaches aim to improve clinical outcomes, enhance patient compliance, reduce dropout rates, and facilitate more tolerable treatment experiences for individuals and couples undergoing infertility care [23,24,25].

Psychosocial well-being is crucial in infertility management, influencing emotional health and treatment outcomes. The stress associated with infertility often leads to decreased QoL and may contribute to treatment discontinuation. Domar et al. (2015) [26] tested a brief cognitive coping and relaxation intervention (CCRI) in women undergoing IVF. Although the intervention did not significantly improve pregnancy rates, it enhanced psychological well-being, reduced anxiety, and was associated with lower treatment dropout rates, supporting the integration of psychosocial care into infertility services [26].

Studies from Central and Eastern Europe reinforce these findings. Wdowiak et al. (2021) [27] demonstrated that women undergoing non-ART treatments report the lowest QoL across physical, psychological, social, and environmental dimensions. Longer treatment duration, higher BMI, and rural residence were associated with poorer outcomes, indicating the complex social and medical factors at play [27]. Similarly, Prémusz et al. (2021) [16] found that emotional and mind–body domains of QoL were most affected by infertility-related stress in women receiving treatment in Southern Hungary. However, social support emerged as a protective factor, mitigating the adverse psychological effects of infertility and improving treatment tolerance [16].

Infertility is a complex condition influenced by a range of biological, behavioural, and psychosocial factors [11,28,29] or pollution. Among these, lifestyle components such as physical activity (PA) and sedentary behaviour (SB) have gained attention due to their potential role in reproductive health [30,31,32].

The 24-h movement guidelines integrate sleep, physical activity, and sedentary behaviour within a single daily framework to define a physically healthy day. Systematic review synthesised existing evidence on how these movement behaviours relate to mental health in non-clinical samples. Significant positive associations were found in 70% of studies involving children, 93% in adolescents, and 89% in adults. Findings suggested that the composition of daily movement behaviours is meaningfully associated with mental health, highlighting important considerations for public health and mental well-being promotion [33].

Evidence also suggests that while moderate PA may have beneficial effects on fertility in both genders, it may also have effects on menstrual cycle irregularities or impaired semen quality. Extremes in either direction—excessive exercise or prolonged inactivity—can be detrimental [34,35,36,37]. Studies highlighted the need for tailored lifestyle interventions that consider individual health status, weight, and baseline activity levels when addressing fertility issues [38].

The tendency towards inactivity increases with age, particularly among women and individuals with lower educational attainment, highlighting the societal and healthcare challenges posed by sedentary lifestyles [39]. This pattern of inactivity is also present among couples undergoing infertility treatment in Hungary [16,40,41]. A recent national survey identified multiple lifestyle-related risk factors in this population, alongside significant psychological distress. In the infertile sample, 27.8% of women and 12.5% of men reported depressive symptoms, while 41% of women and 15% of men experienced elevated stress levels related to fertility difficulties [42].

Despite the potential role of physical activity in supporting reproductive health, the WHO Global Status Report on Physical Activity 2022 points out that targeted, evidence-based recommendations for individuals with infertility still lack [43]. Given the impact of lifestyle on both reproductive outcomes and psychological well-being, integrating structured lifestyle interventions—particularly regular physical activity—into infertility care could be beneficial. Additionally, psychosocial support and counselling remain underutilised, although they may improve treatment adherence and success rates and long-term QoL [44,45,46,47].

Health literacy and fertility awareness influence reproductive outcomes and treatment engagement. Adequate knowledge of reproductive physiology, risk factors, and treatment options is essential for informed decision-making. Kilfoyle et al. (2016) [48] found that higher health literacy is associated with greater awareness of fertility issues, such as recognising the fertile window, and adopting positive health behaviours like prenatal vitamin use. The reviewed studies applied validated instruments, including the REALM, S-TOFHLA, NVS, and SBSQ [48,49,50,51]. Rutherford et al. reported similar findings, showing that women with lower literacy levels had significantly less knowledge of fertility timing, which could compromise effective family planning [52]. Recent data by Zalewska et al. confirmed that women’s knowledge about infertility risks, symptoms, and diagnostics remains limited, regardless of age, education, or personal infertility experience. This underlines the need for population-wide educational interventions [53]. Additionally, Gossett et al. [54] highlighted the importance of numeracy alongside literacy, demonstrating that both are significant predictors of knowledge about age-related fertility decline and ART success rates. These findings suggest that reproductive counselling should be tailored to individual literacy and numeracy capacities to ensure comprehension and engagement [54].

Together, these findings underscore the multifactorial nature of infertility and the importance of integrating lifestyle management, health literacy improvement, and psychosocial support into reproductive healthcare. Previous studies have typically examined these components in isolation; however, the novelty of the present work lies in adopting an integrated approach that simultaneously addresses multiple determinants of infertility-related QoL.

Therefore, we aimed to investigate the associations between the components of 24-h movement behaviour (physical activity, sedentary lifestyle, sleep), health literacy, fertility awareness, and both general and infertility-related QoL in a cross-sectional multi-centre study. The central research question was whether these components can predict quality of life among women living with infertility.

## 2. Materials and Methods

### 2.1. Study Design

This cross-sectional study involved quantitative data collection through self-administered questionnaires. The sample was obtained using a multicentre convenience sampling method. Data collection took place at four institutions in three cities: in Budapest, the capital of Hungary, Pécs and Debrecen, as county seats. All institutions are regional fertility care centres, representing the country’s central, eastern and southern regions.

### 2.2. Recruitment and Eligibility

The target population of the present study consisted of women aged between 18 and 45 years who attended fertility centres for fertility workup or ART. All participants had a previously documented diagnosis of infertility and were consecutively recruited if they had scheduled appointments for ART procedures or pre-treatment consultations at one of the participating fertility centres. During the recruitment process, all women, regardless of the type of infertility (female, male, combined, or unexplained), were invited to participate in the study. Recruitment was continuous and based on the order of arrival at the infertility clinics. The data collection period spanned from 15 October 2024, to 15 January 2025. Exclusion criteria were the presence of major psychiatric disorders and significant physical or mobility impairments that could have interfered with completing the questionnaire or significantly biassed the physical activity-related responses.

**Figure 1:**
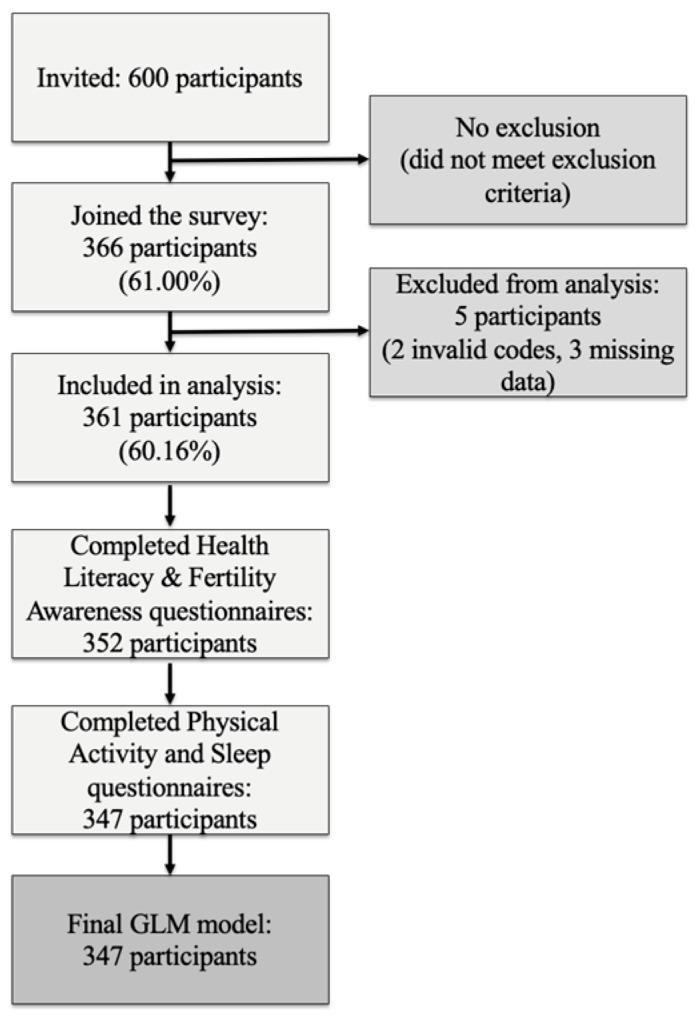
Flow diagram of patient enrolment.

Potential participants were informed about the study via posters in the clinics and verbal invitations from healthcare providers. In total, 600 women were invited to participate in the study. The patient selection process is illustrated in Figure 1.

### 2.3. Data Collection Tools and Procedure

After recruitment, participants received verbal and written information about the study’s aims and procedures. Informed consent was obtained before participation. Patients could join the study by scanning a QR code in the patient information sheet, which directed them to an online questionnaire.

The questionnaire comprised several sections. Sociodemographic characteristics included age, educational attainment, income, and marital status. Health-related questions assessed comorbidities and anthropometric data (height and weight) via self-report. Regarding reproductive history, participants provided information about the duration of their relationship, the length of time spent attempting to conceive, the aetiology of infertility, and the type of fertility treatment.

Health literacy was assessed using the Brief Health Literacy Screening Tool (BRIEF). This self-administered questionnaire evaluates subjective health literacy through four items focusing on potential difficulties in reading and understanding medical documents and processing verbal health-related information. Responses were given on a five-point Likert scale, resulting in total scores between 4 and 20. Scores were categorised as follows: inadequate (4–12), marginal (13–16), and adequate (17–20). The Hungarian validation study reported a Cronbach’s alpha of 0.87, indicating good internal consistency [55]. For this study, the total score was treated as a continuous variable.

Fertility awareness was measured using the Fertility Awareness Survey (FAS). This questionnaire assesses self-reported knowledge about fertility and ART and attitudes toward ART acceptance. It includes 20 control questions addressing various aspects of male and female reproductive health and fertility treatments. Respondents rated their certainty about their knowledge and attitudes supportive or opposing ART on four- and five-point Likert scales. For the analysis, responses were dichotomised into correct (1 point) or incorrect/uncertain (0 points), and the total score was used as a continuous variable. The Hungarian adaptation followed Beaton’s cross-cultural adaptation guidelines [56]. The internal consistency of the Hungarian version reached a Cronbach’s alpha of 0.71, representing the lower acceptable limit [57].

Physical activity was assessed using the Global Physical Activity Questionnaire (GPAQ-H), which evaluates the previous week’s activities according to the 24-h movement behaviour model. The GPAQ-H measures physical activity across three domains: work-related physical activity (WPA), active transport (walking or cycling), and recreational physical activity (RPA). Each category differentiates between vigorous and moderate intensity. Weekly durations of moderate and vigorous activity (MVPA) were calculated in minutes. Additionally, sedentary behaviour (SB) was recorded as the average number of hours spent sitting per day [58].

Sleep was evaluated by asking about the average number of hours of sleep per day. Sleep quality was assessed using the Athens Insomnia Scale (AIS), which comprises eight items: five related to night-time sleep disturbances (such as difficulty falling asleep, waking during the night, early awakening, and sleep quality), and three addressing daytime consequences (mood, energy, and daytime sleepiness). The maximum score is 24, and scores above 10 indicate clinically significant insomnia [59].

Quality of life was assessed using two instruments. General quality of life was measured with the Hungarian version of the World Health Organisation Quality of Life-BREF (WHOQOL-BREF), which evaluates four domains: physical health, psychological well-being, social relationships, and environmental factors. Scores were transformed onto a 0–100 scale, where higher values indicate better quality of life [60].

Fertility-related quality of life was assessed using the Hungarian version of the Fertility Quality of Life questionnaire (FertiQoL). This validated tool measures the quality of life in individuals facing fertility problems across multiple life domains. The Core module assesses emotional well-being, mind–body balance, relationship, and social impact. The optional Treatment module evaluates the experience of medical treatments and their tolerability. Both modules yield scores on a 0–100 scale, with higher scores indicating better fertility-related quality of life [61].

### 2.4. Statistical Analysis

Normality of data distribution was assessed using the Kolmogorov–Smirnov test (*n* = 361). For normally distributed variables, results were presented as mean ± standard deviation (SD), whereas for non-normally distributed variables, medians and interquartile ranges (IQR) were reported. The internal consistency of the scales was evaluated using Cronbach’s alpha, with values above 0.7 considered acceptable [62].

Associations between continuous variables were examined using Spearman’s correlation coefficient. The strength of correlations was categorised according to [63], defining low (r = 0.10), medium (r = 0.20), and high (r = 0.30) correlation strengths.

Multicollinearity among independent variables was tested using the partial eta-squared (η^2^) value. Effect sizes were interpreted based on Cohen’s guidelines: values below 0.01 indicated a small effect, 0.06 a medium effect, and values above 0.14 a large effect [64,65].

To predict fertility-related quality of life (FertiQoL Total), a generalised linear model (GLM) was used due to non-normal data distribution [66]. Model explanatory power was indicated by the R^2^ and adjusted R^2^ values. Partial eta-squared (η^2^) was used to determine the effect size, and 95% confidence intervals were calculated for parameter estimates.

Statistical significance was set at *p* < 0.05 with a 95% confidence interval. The study design and analysis adhered to the Strengthening the Reporting of Observational Studies in Epidemiology (STROBE) guidelines of the Enhancing the Quality and Transparency of Health Research (EQUATOR) Network, and the STROBE checklist for cross-sectional studies was applied [67]. Data analysis was performed using Microsoft Office Excel 2007 and IBM SPSS Statistics version 28.0 (SPSS Inc., Chicago, IL, USA).

## 3. Results

### 3.1. Characteristic of the Sample

The study sample consisted of 361 women undergoing reproductive care. The majority were in their mid-thirties, with a mean age of 34.68 ± 5.03 years. Regarding residence, most participants lived in urban environments (73.7%), while rural residents were underrepresented (26.3%). Marital status data revealed that most were married (86.4%), while 11.4% lived in cohabiting partnerships. The proportion of single participants without a partner was minimal (0.8%), and divorced individuals accounted for only 1.4%.

The educational attainment of the sample was relatively high; 53.2% held tertiary degrees, 43.5% had secondary education, and only 3.3% reported primary education. Participants’ subjective financial situation was generally stable: 66.2% considered their household income average, 25.5% above average, and 8.3% reported below-average income. Based on anthropometric measures, the average BMI was 25.12 ± 5.15 kg/m^2^.

Regarding reproductive health, most participants were in long-term relationships, averaging 7.99 ± 4.69 years. The mean time spent attempting to conceive was 40.17 ± 30.09 months, approximately 3 years and 4 months. Some participants had already experienced parenthood, with 14.5% having one child and 6.6% having two children. Many underwent their second or third treatment cycle, with a mean of 2.34 ± 1.55 cycles. Treatment types included hormonal therapy, intrauterine insemination (IUI), and, primarily, in vitro fertilisation (IVF), which was reported by 70.9% of the participants.

Regarding infertility diagnosis, female factor infertility was identified in 34.1% of cases, male factor in 15.8%, and combined causes in 19.1%. In 26.9% of cases, the cause remained unexplained, while 4.2% were still undergoing diagnostic procedures. A small proportion of the sample (6.4%) had not yet started fertility treatment and were in the assessment phase Table 1.

### 3.2. Fertility Awareness and Health Literacy

Based on the Fertility Awareness Survey, 77.8% of participants (*n* = 274) self-reported having either “acceptable” or “reliable” knowledge regarding fertility, suggesting that nearly four out of five respondents perceive themselves as adequately informed. Regarding knowledge of ART, a similar pattern emerged, with 75.3% of participants (*n* = 265) reporting “acceptable” or “reliable” knowledge of the procedures. In terms of attitudes towards ART, 36.1% of participants (*n* = 127) expressed a “supportive” or “highly supportive” stance. This indicates that approximately one-third of the respondents hold a clearly positive view of ART, while the majority remain neutral, and a small minority (*n* = 3; 0.9%) reported negative attitudes. When examining objective knowledge scores, the average score on the Fertility Awareness Survey was 9.69 ± 2.54 out of a maximum of 20, corresponding to 48.5% of the possible total. This highlights a discrepancy between self-perceived and actual knowledge levels, suggesting room for improvement in fertility-related education.

Health literacy, measured by the BRIEF scale, showed a mean score of 9.22 ± 2.87 out of 20, representing 46.1% of the maximum score, reflecting a moderate health literacy level within the sample Table 2.

### 3.3. 24-h Movement Behaviour Patterns

According to the GPAQ-H, work-related PA showed considerable variability within the sample. The mean weekly vigorous work-related PA was 213.7 ± 528.6 min, with a median of 0 min per week, indicating that many participants did not engage in this type of activity. Similarly, moderate work-related PA averaged 346.5 ± 648.8 min per week, with a median of 0, suggesting limited prevalence across the sample. MVPA reached a weekly mean of 773.9 ± 1515.0 min, but the median remained low at 30.0 min, reflecting substantial heterogeneity. Active transport, such as walking or cycling for commuting purposes, was more common, with a mean of 196.0 ± 397.0 min per week and a median of 60.0 min. In the domain of recreational PA, weekly vigorous activity averaged 58.8 ± 154.5 min, and moderate activity averaged 90.2 ± 162.0 min, with medians of 0 and 30.0 min, respectively. The recreational MVPA was 207.7 ± 403.7 min per week, with a median of 70.0 min, indicating that regular recreational activity was limited to a subset of participants. Overall, total MVPA across all domains averaged 1177.6 ± 1789.5 min per week, with a median of 450.0 min. The lower quartile engaged in less than 100 min of MVPA per week, while the upper quartile exceeded 1290 min, highlighting significant variability in PA patterns. Sedentary behaviour (SB) averaged 449.7 ± 225.1 min per day (median 450.0), indicating that participants typically spent around 7.5 h per day in sedentary activities.

Participants reported an average sleep duration of 7.21 ± 1.03 h per night, which aligns with general sleep recommendations for adults. Based on the Athens Insomnia Scale (AIS), the mean total score was 3.78 ± 3.31, indicating overall good self-reported sleep quality, as values below 10 do not suggest clinical insomnia. Nevertheless, 20 participants (5.0%) reached or exceeded the threshold of 10 points, placing them in the insomnia risk group and highlighting the need for targeted sleep quality monitoring within this population.

### 3.4. General and Infertility-Specific Quality of Life Assessment

According to the FertiQoL results, women living with infertility reported a moderately favourable infertility-related quality of life, though substantial variation was observed across subscales. The average perceived health score related to infertility was 69.99 ± 18.66, while the global quality of life score was 64.69 ± 24.53. These findings align with the general WHOQOL-BREF results but suggest that women perceive their infertility-related health more negatively than their general health, with large standard deviations reflecting diverse personal experiences and coping strategies.

The highest FertiQoL score appeared in the relational domain (77.61 ± 16.93), indicating stable partner support despite fertility challenges. Conversely, the mind–body (58.42 ± 23.70) and emotional (60.64 ± 22.86) domains showed lower scores, highlighting the substantial psychological and physical burden of infertility. The social dimension reflected moderate satisfaction (64.88 ± 22.00), though the variability suggests differences in perceived social support and experiences of stigma.

The overall Core Score was 65.39 ± 17.47, indicating moderate infertility-specific quality of life. Regarding treatment, the tolerability subscale (69.04 ± 20.47) suggested that most women could cope with the demands of assisted reproductive technologies. In contrast, the environmental subscale (44.97 ± 12.74) indicated dissatisfaction with healthcare system factors such as access to information and treatment conditions. The total treatment score was 54.60 ± 9.89, and the FertiQoL total score was 62.31 ± 13.75, reflecting a moderate impact of infertility on quality of life, with emotional, physical, and systemic factors causing considerable strain despite strong partner support Table 3.

### 3.5. Health Awareness and 24-h Movement Behaviour

Spearman’s rank correlation analysis was used to examine the associations between health literacy (BRIEF), fertility awareness (FAS), various dimensions of physical activity (work-related, recreational, total MVPA), sedentary behaviour, and sleep parameters. Health literacy was negatively correlated with work-related MVPA (r = −0.133; *p* = 0.012) and showed a weak negative association with total MVPA (r = −0.103; *p* = 0.050), indicating that participants with higher health literacy reported lower levels of occupational physical activity. No significant correlations were found between health literacy and other PA domains, sedentary behaviour, or sleep parameters.

Fertility awareness was positively associated with recreational physical activity (r = 0.156; *p* = 0.003), while no significant associations were observed with other PA forms, sedentary time, or sleep duration and quality (sleep disturbances) Table 4.

### 3.6. General and Infertility-Specific Quality of Life and 24-h Movement Behaviour

Analysis of the WHOQOL-BREF dimensions showed that recreational physical activity (RPA) was positively associated with several QoL variables. The strongest correlation was with self-rated health (r = 0.197; *p* ≤ 0.001), followed by environmental factors (r = 0.191; *p* ≤ 0.001), psychological well-being (r = 0.177; *p* = 0.001), physical health (r = 0.169; *p* = 0.002), social relationships (r = 0.145; *p* = 0.007), and overall quality of life (r = 0.136; *p* = 0.011). Total MVPA was also associated with psychological well-being (r = 0.139; *p* = 0.024). Sleep duration was moderately correlated with physical health (r = 0.319; *p* ≤ 0.001) and psychological well-being (r = 0.210; *p* ≤ 0.001), and showed weaker associations with self-rated health (r = 0.107; *p* = 0.048) and social relationships (r = 0.126; *p* = 0.020). Sedentary behaviour was negatively associated with psychological well-being (r = −0.109; *p* = 0.042) and social relationships (r = −0.118; *p* = 0.027). The strongest negative correlations were between sleep quality (AIS score) and all WHOQOL dimensions, particularly physical health (r = −0.542; *p* ≤ 0.001), psychological well-being (r = −0.476; *p* ≤ 0.001), and social relationships (r = −0.292; *p* ≤ 0.001).

Regarding infertility-specific FertiQoL, recreational PA was positively associated with self-rated health (r = 0.236; *p* ≤ 0.001) and overall quality of life (r = 0.147; *p* = 0.006). No significant associations were found between RPA and other FertiQoL domains. Sedentary behaviour showed negative associations with emotional (r = −0.162; *p* = 0.002), mind–body (r = −0.178; *p* = 0.001), social (r = −0.106; *p* = 0.047), FertiQoL Core (r = −0.162; *p* = 0.002), and FertiQoL Total (r = −0.150; *p* = 0.005) scores.

Sleep quality, measured by AIS, showed significant negative correlations with nearly all FertiQoL domains, including health (r = −0.242; *p* ≤ 0.001), overall quality of life (r = −0.246; *p* ≤ 0.001), emotional (r = −0.334; *p* ≤ 0.001), mind–body (r = −0.323; *p* ≤ 0.001), partner relationship (r = −0.124; *p* = 0.020), social (r = −0.300; *p* ≤ 0.001), treatment tolerability (r = −0.205; *p* ≤ 0.001), treatment environment (r = −0.126; *p* = 0.020), FertiQoL Core (r = −0.339; *p* ≤ 0.001), and FertiQoL Total (r = −0.330; *p* ≤ 0.001). Higher AIS scores indicate poorer sleep; thus, these results suggest that better sleep quality is consistently associated with better infertility-specific quality of life, particularly in emotional and mind–body domains. However, correlations below r = 0.150 should be interpreted cautiously; avoid overclaiming clinical relevance Tables S1 and S2.

### 3.7. Prediction of Infertility-Specific Quality of Life by 24-h Movement Behaviour and Health Awareness

We expected higher PA and awareness, coupled with lower sedentary time and better sleep, to be associated with improved quality of life, while negative behavioural patterns (e.g., prolonged sitting, poor sleep quality) would correlate with lower well-being. A Generalised Linear Model (GLM) was used to predict the FertiQoL Total score. Independent variables included work-related MVPA, recreational MVPA, total MVPA (GPAQ), sedentary time (GPAQ), sleep quality (AIS), health literacy (BRIEF), and fertility awareness (FAS). Since the three PA components (work, recreational, and total MVPA) were not significantly associated with the dependent variable, they were removed from the final model.

The final model included sleep quality (AIS), sedentary time (GPAQ), health literacy (BRIEF), and fertility awareness (FAS). Results indicated that sleep quality was a significant negative predictor of infertility-specific quality of life (B = −1.29; β = −0.469; *p* ≤ 0.001; η^2^ₚ = 0.100), suggesting that poorer sleep quality substantially reduces FertiQoL scores. Sedentary time was also negatively associated with FertiQoL (B = −0.008; β = −0.079; *p* = 0.008; η^2^ₚ = 0.020), supporting the detrimental impact of prolonged inactivity on psychological well-being. Health literacy emerged as a positive predictor (B = 0.63; β = 0.285; *p* = 0.016; η^2^ₚ = 0.017), indicating that higher health knowledge may improve psychosocial adaptation in infertility treatment. Conversely, higher fertility awareness was negatively associated with FertiQoL (B = −0.67; β = −0.310; *p* = 0.016; η^2^ₚ = 0.017), potentially reflecting the anxiety-provoking effects of greater awareness of risks and treatment uncertainties.

The model explained 15.5% of the variance in FertiQoL Total (R^2^ = 0.155; F = 8.881; *p* < 0.001). According to conventional social science thresholds, the partial eta-squared values indicated small to medium effect sizes [64,65] (Table 5).

## 4. Discussion

This study investigated the associations between 24-h movement behaviour components (physical activity, sedentary behaviour, sleep), health literacy, fertility awareness, and infertility-specific quality of life in women undergoing fertility treatment. The findings revealed that recreational physical activity and better sleep quality might be positively associated with both general and infertility-related quality of life. In contrast, sedentary time and sleep disturbances might show negative correlations. Furthermore, the generalised linear model identified sleep quality, sedentary behaviour, health literacy, and fertility awareness as significant predictors of infertility-specific quality of life.

In line with previous findings, HL has been shown to play a pivotal role in promoting health behaviours and preventing chronic diseases. A systematic review including 22 studies found that higher HL levels were significantly associated with greater PA engagement in 18 studies, regardless of PA type [68]. An Australian study using the BRIEF tool confirmed HL as a predictor of PA, dietary behaviours, and healthcare use [69], while an extensive Danish cohort study demonstrated that HL mediated the relationship between educational attainment and health behaviours, including PA [70]. Our findings partially support this evidence: while no significant association was found between HL and recreational PA (RPA), HL was negatively correlated with work-related PA (ρ = −0.133; *p* = 0.012), potentially reflecting a higher prevalence of sedentary occupations among individuals with higher HL. Similarly, the association between HL and total MVPA was marginal (ρ = −0.103; *p* = 0.050). It is possible that the inherently inactive nature of our sample limited our ability to detect a direct association.

Regarding fertility awareness, our results indicated a significant positive association with recreational PA (ρ = 0.156; *p* = 0.003), while no association was found with work-related or total MVPA. These findings align with prior evidence suggesting that individuals who are more conscious of their fertility tend to adopt healthier lifestyle practices. A Polish study by Chawłowska et al. mentioned the same association. However, it revealed that young women’s fertility awareness was incomplete, particularly concerning lifestyle factors such as nutrition, sleep, and physical exertion [71]. Our findings are also consistent with the goals of fertility-focused lifestyle interventions, such as the PreLiFe programme, a mobile-based lifestyle intervention supporting PA, nutrition, and mindfulness among couples undergoing IVF [72]. This underscores the relevance of personalised, multicomponent interventions promoting healthy lifestyle patterns in fertility care.

Our findings also suggest that RPA is positively associated with multiple dimensions of quality of life. Low but significant correlations were observed with physical health (r = 0.169; *p* ≤ 0.002), psychological well-being (r = 0.177; *p* ≤ 0.002), and other quality of life components, indicating that regular recreational exercise may contribute to better overall well-being. No significant association was found between work-related PA and quality of life, while total MVPA showed a weak positive correlation only with psychological well-being (r = 0.139; *p* = 0.024). In contrast, sedentary behaviour has played a more pronounced role in shaping the observed outcomes. It was negatively associated with psychological well-being (r = −0.109; *p* = 0.042) and social relationships (r = −0.118; *p* = 0.027), supporting previous findings [16] that prolonged sitting may be detrimental to mental health and social connectedness. Sleep duration was positively associated with four quality of life domains, especially physical health (r = 0.319; *p* ≤ 0.001), while sleep quality problems were inversely correlated with all QoL dimensions, highlighting the role of restorative sleep in subjective well-being.

These results are consistent with broader literature on life satisfaction, a cognitive appraisal of one’s overall quality of life that is strongly linked to mental and physical health outcomes and mortality and morbidity [73]. In a previous longitudinal European study, recreational outdoor PA was found to significantly increase life satisfaction among reproductive-age women, with participants engaging in outdoor activities being 21.4% more likely to report being “very satisfied” with life compared to inactive individuals. This effect remained significant in middle-aged women (35–49 years), supporting the specific benefits of green-space PA on both physical and psychological health. Our current findings align with these observations, emphasising that recreational PA—particularly when performed outdoors—can enhance various aspects of quality of life, including mental well-being.

Our findings indicate that recreational physical activity (RPA) was positively associated with several dimensions of infertility-specific quality of life. Positive associations were observed with health perception (r = 0.236) and general quality of life (r = 0.147), while no significant relationships were found with work-related or total MVPA. This suggests that the beneficial effects are primarily linked to recreational forms of movement.

Sedentary behaviour (SB) showed significant negative correlations with multiple FertiQoL aspects, especially psychological components such as mind–body well-being and emotional functioning. These results support the growing body of evidence indicating that prolonged sedentary time can negatively affect psychological well-being in the general population Mező [74,75,76,77] and in individuals undergoing infertility treatment.

Sleep quality also demonstrated significant negative associations with all FertiQoL dimensions (e.g., FertiQoL Core: r = −0.339; *p* ≤ 0.001), while longer sleep duration was positively correlated with life quality outcomes (e.g., quality of life: r = 0.112; *p* = 0.037). These findings highlight the importance of sleep in the subjective experience of infertility-related quality of life.

Finally, although the 24-HMB concept could not be fully confirmed and physical activity was not a significant predictor in the model, its somatic and psychological relevance was demonstrated. Moderate physical activity may reduce oxidative stress by enhancing mitochondrial function and antioxidant defences, whereas excessive exercise may increase oxidative stress and potentially impair reproductive function [74,75,76]. In addition to physical activity, some authors have also highlighted the importance of nutrition, particularly the role of the Mediterranean diet [77,78,79].

Our results are consistent with recent randomised controlled trials addressing the impact of lifestyle and psychological interventions on reproductive health. Szigeti et al. tested the effectiveness of the Mind/Body Program for Infertility (MBPI) in a Hungarian clinical sample [20]. Their 10-week structured intervention, based on cognitive behavioural therapy and stress management, significantly reduced trait anxiety. However, no significant differences between intervention and control groups in infertility-specific quality of life (FertiQoL), other psychological outcomes, or medical endpoints were found. Nevertheless, general well-being improved in both groups, underscoring the importance of health-promoting support in infertility care.

These findings parallel our study. RPA was associated with better infertility-specific quality of life, while SB and sleep quality were linked to psychological and general well-being outcomes. Physical inactivity and poor sleep quality may adversely affect life quality in women with infertility, particularly regarding psychological aspects. Our results reinforce the need for structured support targeting movement and rest—whether through lifestyle programmes or psychological interventions—to improve quality of life in this vulnerable population [20].

In a recent study, Moutzouroulia et al. [80] examined factors affecting mental health and quality of life in women undergoing in vitro fertilisation (IVF). Their regression analysis revealed that pregnancy status significantly influenced emotional (β = 0.018; *p* = 0.050) and mind–body dimensions (β = 0.030; *p* = 0.002), while marital status was associated with higher social dimension scores (β = 0.019; *p* = 0.016). Additionally, women with higher educational attainment reported better outcomes in the environmental domain (β = 0.011; *p* = 0.041). Longer duration of infertility and higher scores on the Fertility Problem Inventory (FPI) were linked to lower tolerability scores (β = −0.005; *p* = 0.050; β = −0.002; *p* = 0.016) [80].

Similarly to Moutzouroulia et al. (2025) [80], our results highlight the multidimensional factors influencing infertility-related quality of life. In our study, sleep quality, SB, health literacy, and fertility awareness were significant predictors of FertiQoL scores, with sleep problems and sedentary behaviour associated with lower well-being. In contrast, higher health literacy showed a positive association. Conversely, greater fertility awareness was linked to lower quality of life, potentially due to the psychological burden of increased knowledge. The paradoxical negative association between fertility awareness and infertility-related QoL may reflect the psychological costs of risk-focused knowledge. Evidence from similar fields, as prenatal screening and ovarian reserve testing, suggests that more–pr poorly tailored information can heighten anxiety and distress rather than reduce it under low perceived control. These patterns underscore that the quality and framing of knowledge and accompanying counselling are critical moderators of its emotional impact [81,82,83,84].

Our findings underscore the importance of psychological support and adaptive coping strategies in improving quality of life among women undergoing infertility treatment [80]. Our predictive model identified sleep quality, sedentary behaviour, health literacy, and fertility awareness as significant factors influencing infertility-specific quality of life. Poorer sleep quality and longer sedentary time were associated with lower FertiQoL scores, while higher health literacy was linked to better outcomes. Interestingly, higher fertility awareness was negatively related to infertility-specific quality of life, suggesting that increased awareness might heighten anxiety due to a more accurate perception of treatment risks and uncertainties. None of the physical activity components were significant predictors in the model.

Although our predictive model did not confirm our original hypothesis—that higher fertility awareness would lead to more recreational physical activity and thereby improve quality of life—the lack of association may be explained by the sample’s overall inactivity and the overriding influence of other variables. Nevertheless, we are convinced that combining educational strategies to enhance awareness with lifestyle interventions promoting physical activity could improve infertility-specific quality of life, which is essential for maintaining well-being and perseverance throughout often years-long treatment processes. This, in turn, may help couples reach the cumulative pregnancy rates necessary for treatment success. While the benefits of physical activity are typically discussed in terms of direct effects on clinical outcomes, its supportive role in sustaining mental health and treatment adherence deserves greater emphasis. What we consider the most novel and significant contribution of our work is the emphasis on the supportive role of non-exhaustive physical activity in enhancing infertility-specific quality of life. We continue to recommend incorporating regular, moderate-intensity physical activity for all couples undergoing fertility treatment as a means to support their psychological resilience and long-term engagement in the process.

### Limitations

This study has several methodological and interpretive limitations that should be considered when contextualising the findings. Firstly, convenience sampling limits the generalisability of the results, as the participants do not represent the broader population of Hungarian women living with infertility. Additionally, data collection was based on self-administered questionnaires, which may introduce bias due to participants’ perceptions, response styles, or current psychological states. Some correlations found were very low (r < 0.20); caution is needed in interpretation.

Another limitation is that the current analysis focused exclusively on female participants, even though the broader study included male respondents as well. Therefore, the findings do not reflect the experiences or quality of life of men living with infertility.

Furthermore, while the predictive model considered specific behavioural and psychosocial factors, several other relevant covariates may influence infertility-specific quality of life but were not included in the current analysis. These may consist of educational attainment, socioeconomic status (SES), the type and stage of fertility treatment, underlying infertility aetiology, and previous loss experiences. The use of complementary and alternative therapies may also play a role. In addition, dyadic relationship functioning, such as partner support and couple dynamics, is known to affect quality of life during infertility treatment significantly, but this was not the focus of the present study.

Future research should address these gaps by including more diverse samples, incorporating longitudinal designs, and examining a broader range of psychosocial and contextual factors to understand the multifaceted experience of infertility better.

## 5. Conclusions

These findings highlight the role of 24-h movement behaviour and health awareness in improving the quality of life among women with infertility. The study supports the need for tailored, multi-component lifestyle interventions to promote integrated physical, mental, and social well-being, particularly during pre-rehabilitation.

Specifically, recreational physical activity was positively associated with general and infertility-specific quality of life. In contrast, prolonged sedentary behaviour and poor sleep quality were linked to lower well-being across multiple domains. Health literacy emerged as a positive predictor of infertility-related quality of life, suggesting that enhancing patients’ knowledge and competence may improve psychosocial adaptation.

These results underscore the importance of integrating lifestyle counselling—focused on increasing physical activity, reducing sedentary behaviour, and improving sleep quality—into infertility care. Additionally, educational and psychological support should be carefully tailored to avoid information overload and to balance awareness with emotional coping strategies. A patient-centred, holistic approach may improve treatment adherence and long-term psychosocial well-being. However, the cross-sectional nature of this study warrants a tempered interpretation of these recommendations.

## Figures and Tables

**Table 1:** Socio-demographic, Anthropometric, Clinical and Reproductive Health Characteristics of the Sample (*n* = 361).

Sociodemographic and Anthropometric Characteristics		
	*n*	%
Type of residence		
Capital city	86	23.8%
County seat	52	14.4%
City	128	35.5%
Village	95	26.3%
Level of education		
Primary	12	3.3%
Secondary	157	43.5%
Tertiary	192	53.2%
Household income		
Below average	30	8.3%
Average	239	66.2%
Above average	92	25.5%
Age	Mean	SD
Years	34.68	5.03
Anthropometrics	Mean	SD
Height (cm)	165.66	6.29
Weight (kg)	68.91	14.39
BMI (kg/m^2^)	25.12	5.15
Clinical and Reproductive Health Characteristics		
Childbearing history	Mean	SD
In relationship (years)	7.99	4.69
Attempting to conceive (month)	40.17	30.09
Treatment cycles (n)	2.34	1.55
Own children	n	%
0	285	78.9
1	52	14.5
2	24	6.6
Cause of infertility	n	%
Female factor	123	34.1%
Male factor	57	15.8%
Combined factor	69	19.1%
Unknown	97	26.9%
Diagnostic process ongoing	15	4.2%
Type of Fertility Treatment		
No treatment yet	23	6.4%
Tubal flushing	17	4.7%
Hormonal therapy	26	7.2%
IUI	39	10.8%
IVF/ICSI/FET	256	70.9%

Body Mass Index (BMI); Frozen embryo transfer (FET) In vitro fertilisation (IVF); Intracytoplasmic sperm injection (ICSI); Intrauterine insemination (IUI).

**Table 2:** General Health Literacy, Fertility and Assisted Reproductive Technology (ART) Knowledge, Self-Perceived Fertility Awareness, and Attitudes Toward ART Among Women Living with Infertility (*n* = 352).

Scales	Responses	*n*	%
Fertility Knowledge	Does not have knowledge	18	5.1%
	Some knowledge	60	17.0%
	Acceptable knowledge	181	51.4%
	Reliable knowledge	93	26.4%
ART Knowledge	Does not have knowledge	20	5.7%
	Some knowledge	67	19.0%
	Acceptable knowledge	177	50.3%
	Reliable knowledge	88	25.0%
ART Attitude	Strongly opposes	4	1.1%
	Opposes	54	15.3%
	Neither supports nor opposes	167	47.4%
	Supports	124	35.2%
	Strongly supports	3	0.9%
FAS Total Score	Max 20 points	Mean	9.69
		SD	2.54
BRIEF Total Score	Max 20 points	Mean	9.22
		SD	2.87

Assisted Reproductive Technology (ART); Brief Health Literacy Screening Tool (BRIEF); Fertility Awareness Survey (FAS).

**Table 3:** General and Infertility-Specific Quality of Life Assessment Based on WHOQOL-BREF and FertiQoL Questionnaires (*n* = 354).

WHOQOL-BREF		Mean	SD
SRH		73.71	15.13
QoL		65.45	18.60
WHOQOL-BREF Domains	Physical	79.36	12.75
	Psychological	67.85	16.46
	Social	70.57	20.22
	Environmental	73.16	14.87
FertiQoL		Mean	SD
SRH		69.99	18.66
QoL		64.69	24.53
FertiQoL Domains	Emotional	60.64	22.86
	Mind–Body	58.42	23.70
	Relational	77.61	16.93
	Social	64.88	22.00
Core Scale		65.39	17.47
	Environment (*n* = 342)	44.97	12.74
	Tolerability (*n* = 342)	69.04	20.47
Treatment Scale (*n* = 342)		54.60	9.89
FertiQoL Total		62.31	13.75

Fertility Quality of Life questionnaire (FertiQoL); Quality of Life (QoL); Self-Rated Health (SRH); World Health Organization Quality of Life–BREF questionnaire (WHOQOL-BREF).

**Table 4:** Associations Between Health Literacy (BRIEF) and Fertility Awareness (FAS) and 24-h Movement Behaviour Components: Spearman’s Rank Correlation Analysis (*n* = 347).

24-HMB		BRIEF	FAS
W MVPA	r	−0.133 *	−0.020
	*p*	0.012	0.709
R MVPA	r	0.066	0.156 **
	*p*	0.212	0.003
Total MVPA	r	−0.103 *	0.068
	*p*	0.050	0.207
SB	r	0.072	0.062
	*p*	0.174	0.244
Sleep time	r	0.034	0.013
	*p*	0.525	0.805
AIS	r	−0.095	0.071
	*p*	0.073	0.186

* *p* ≤ 0.05 and ** *p* ≤ 0.01; 24-h Movement Behaviour (24-HMB); Brief Health Literacy Screening Tool (BRIEF); Fertility Awareness Survey (FAS); Work-Related Moderate-to-Vigorous Physical Activity (W MVPA); Recreational Moderate-to-Vigorous Physical Activity (R MVPA); Total Moderate-to-Vigorous Physical Activity (Total MVPA); Sedentary Behaviour (SB); Athens Insomnia Scale (AIS).

**Table 5:** Prediction of Infertility-Specific Quality of Life (FertiQoL Total) Using a Generalised Linear Model (GLM) Based on 24-h Movement Behaviour Components and Awareness Factors (*n* = 347).

	B	SE	β	t	*p*	η^2^p
Model (Intercept)	67.354	5.107		13.188	≤0.001	
Sleep Quality (AIS)	−1.29	0.209	−0.469	−6.149	≤0.001	0.100
Sedentary Behaviour (GPAQ)	−0.008	0.003	−0.079	−2.652	0.008	0.020
*Health Literacy* (BRIEF)	0.63	0.261	0.285	2.420	0.016	0.017
Fertility Awareness (FAS)	−0.67	0.277	−0.310	−2.420	0.016	0.017

R^2^ = 0.155; F = 8.881; *p* < 0.001. Variables not retained in the final model: Work-related MVPA; Recreational MVPA; Total MVPA; Athens Insomnia Scale (AIS); Brief Health Literacy Screening Tool (BRIEF); Fertility Awareness Survey (FAS); Global Physical Activity Questionnaire (GPAQ).

## Data Availability

The dataset supporting the conclusions of this article is not publicly available but is available from the corresponding author on reasonable request.

## Supplementary Materials

The following supporting information can be downloaded at: https://www.mdpi.com/article/10.3390/jcm14186552/s1, Table S1. Associations Between General Quality of Life (WHOQOL-BREF) and 24-Hour Movement Behaviour Components: Spearman’s Rank Correlation Analysis (*n* = 347); Table S2. Associations Between Infertility-Specific Quality of Life (FertiQoL) and 24-Hour Movement Behaviour Components: Spearman’s Rank Correlation Analysis (*n* = 347).

## Abbreviations

The following abbreviations are used in this manuscript:

AIS	Athens Insomnia Scale
ART	Assisted Reproductive Technologies
BRIEF	Brief Health Literacy Screening Tool
EQUATOR	Enhancing the Quality and Transparency of Health Research
FAS	Fertility Awareness Survey
FertiQoL	Fertility Quality of Life Questionnaire
FPI	Fertility Problem Inventory
GBD	Global Burden of Disease Study
GLM	Generalised Linear Model
GPAQ-H	Global Physical Activity Questionnaire–Hungarian Version
IUI	Intrauterine Insemination
IVF	In Vitro Fertilisation
MBPI	Mind/Body Program for Infertility
MVPA	Moderate and Vigorous Physical Activity
RPA	Recreational Physical Activity
SB	Sedentary Behaviour
SD	Standard Deviation
STROBE	Strengthening the Reporting of Observational Studies in Epidemiology
WHOQOL-BREF	World Health Organisation Quality of Life-BREF
WPA	Work-related Physical Activity
