# Supplementary material for: 24-Hour Movement Behaviour and Health Awareness as Possible Predictors of Infertility-Related Quality of Life
Source: J Clin Med. 2025 Sep 17;14(18):6552. doi: 10.3390/jcm14186552 (PMC12470367; doi:10.3390/jcm14186552)
Supplement: Supplementary file 1 [file jcm-14-06552-s001.zip › jcm-3808590-supplementary.pdf]

Supplementary Materials

Table S1. Associations Between General Quality of Life (WHOQOL-BREF) and 24-Hour Movement Behaviour Components: Spearman's Rank Correlation Analysis (N=347)

WHOQOL	*W MVPA*		*R MVPA*		*T MVPA*		*SB*		*Sleep time*		*AIS*	
	r	*p*	*r*	*p*	*r*	*p*	*r*	*p*	*r*	*p*	*r*	*p*
QoL	-0.092	0.087	**0.136***	**0.011**	0.015	0.853	-0.010	0.852	0.033	0.541	**-0.318****	**≤0.001**
SRH	-0.100	0.062	**0.197****	**≤0.001**	0.087	0.783	-0.017	0.747	**0.107***	**0.048**	**-0.293****	**≤0.001**
Physical	0.002	0.963	**0.169****	**0.002**	**0.121***	**0.107**	-0.014	0.795	**0.319****	**≤0.001**	**-0.542****	**≤0.001**
Psychological	0.035	0.518	**0.177****	**0.001**	**0.139****	**0.024**	**-0.109***	**0.042**	**0.210****	**≤0.001**	**-0.476****	**≤0.001**
Social	0.083	0.121	**0.145****	**0.007**	**0.067**	**0.009**	**-0.118***	**0.027**	**0.126***	**0.020**	**-0.292****	**≤0.001**
Environmental	-0.012	0.821	**0.191****	**≤0.001**	0.032	0.213	-0.008	0.884	0.100	0.064	**-0.249****	**≤0.001**

*$p \le 0.05$ and **$p \le 0.01$

Athens Insomnia Scale (AIS), Quality of Life (QoL), Recreational Moderate-to-Vigorous Physical Activity (R MVPA), Self-Rated Health (SRH), Total Moderate-to-Vigorous Physical Activity (T MVPA), Work Moderate-to-Vigorous Physical Activity (W MVPA), World Health Organization Quality of Life Questionnaire – Brief Version (WHOQOL-BREF)

Table S2. Associations Between Infertility-Specific Quality of Life (FertiQoL) and 24-Hour Movement Behaviour Components: Spearman's Rank Correlation Analysis (N=347)

FertiQoL	W MVPA		R MVPA		T MVPA		SB		Sleep time		AIS	
	r	p	r	p	r	p	r	p	r	p	r	p
SRH	0,031	0,560	**0,236****	**≤0,001**	0,069	0,020	-0,091	0,086	0,037	0,497	**-0,242****	**≤0,001**
QoL	0,005	0,920	**0,147****	**0,006**	0,007	0,193	-0,082	0,122	**0,112***	**0,037**	**-0,246****	**≤0,001**
Emotional	0,012	0,824	0,036	0,500	0,004	0,899	**-0,162****	**0,002**	0,068	0,210	**-0,334****	**≤0,001**
Mind-Body	-0,005	0,921	0,053	0,323	0,059	0,934	**-0,178****	**0,001**	0,044	0,420	**-0,323****	**≤0,001**
Relational	-0,001	0,981	0,075	0,159	0,035	0,264	-0,087	0,101	0,037	0,495	**-0,124***	**0,020**
Social	0,030	0,575	0,021	0,693	0,021	0,513	**-0,106***	**0,047**	0,049	0,364	**-0,300****	**≤0,001**
Core Scale	0,009	0,873	0,047	0,375	-0,094	0,689	**-0,162****	**0,002**	0,064	0,234	**-0,339****	**≤0,001**
Environment	-0,068	0,210	-0,061	0,262	0,008	0,084	**0,142****	**0,009**	-0,020	0,713	0,083	0,125
Tolerability	0,008	0,886	0,003	0,950	-0,056	0,877	-0,104	0,055	0,031	0,576	**-0,205****	**≤0,001**
Treamant Scale	-0,036	0,504	-0,038	0,483	0,005	0,303	0,000	0,997	0,000	0,996	**-0,126***	**0,020**
FertiQoL Total	-0,002	0,969	0,031	0,565	0,010	0,930	**-0,150****	**0,005**	0,047	0,387	**-0,330****	**≤0,001**

*$p \leq 0.05$ and **$p \leq 0.01$

Athens Insomnia Scale (AIS), Fertility Quality of Life Questionnaire (FertiQoL), Quality of Life (QoL), Recreational Moderate-to-Vigorous Physical Activity (R MVPA), Self-Rated Health (SRH), Total Moderate-to-Vigorous Physical Activity (T MVPA), Work Moderate-to-Vigorous Physical Activity (W MVPA
